# Impact of long‐term maternal high‐fat, high‐sugar feeding on gastric vagal afferent responses and feeding behaviour in mouse offspring

**DOI:** 10.1113/EP093108

**Published:** 2025-11-06

**Authors:** Georgia S. Clarke, Hui Li, Elaheh Heshmati, Kelly M. Gembus, Stephanie E. O'Hara, Lisa M. Nicholas, Kathryn L. Gatford, Amanda J. Page

**Affiliations:** ^1^ School of Biomedicine University of Adelaide Adelaide SA Australia; ^2^ Nutrition, Diabetes & Gut Health, Lifelong Health Theme South Australian Health and Medical Research Institute Adelaide SA Australia; ^3^ Robinson Research Institute University of Adelaide Adelaide SA Australia; ^4^ Adelaide Centre for Epigenetics University of Adelaide Adelaide SA Australia

**Keywords:** developmental programming, food intake, high‐fat high‐sugar diet, mouse, offspring, vagal afferent

## Abstract

Poor maternal nutrition and excessive gestational weight gain predict future development of obesity in offspring. Preclinically, maternal obesity induced by a high‐fat, high‐sugar diet (HFHSD) induces hyperphagia and obesity in offspring. We hypothesized that this might, in part, reflect reduced peripheral gastric vagal afferent (GVA) satiety signalling. Female Glu Venus mice were fed a standard laboratory diet (SLD) or HFHSD for 11 weeks before mating and throughout pregnancy and lactation. Offspring were weaned onto a SLD, then housed in metabolic cages at 6–7 weeks old to assess feeding behaviour. In vitro single‐fibre GVA recordings were conducted on tissue collected from 8‐week‐old mice. Before mating, HFHSD dams were 13% heavier, with 66% higher relative fat mass compared with SLD dams. Maternal diet had no impact on total food intake or offspring weight. Meal size during the light phase was 14% larger in HFHSD than control offspring. Meal duration was longer in HFHSD than control offspring of both sexes across 24 h and the dark phase, and in females during the light phase. HFHSD offspring ate fewer meals than control offspring across all time periods. Tension‐sensitive GVAs responded less to stretch in male, but not female, HFHSD than SLD offspring. Mucosal GVA responses to mucosal stroking were unaffected by maternal diet or offspring sex. In conclusion, exposure in utero and during lactation to elevated maternal adiposity and maternal HFHSD consumption induces male‐specific programming of reduced GVA responses to stretch. Meal size was increased in both sexes only during the light phase, suggesting programming of other appetite‐regulatory pathways by this exposure.

## Abstract (highlights)

- **What is the central question of this study?**
  Poor maternal nutrition and excessive gestational weight gain program development of obesity in offspring. Gastric vagal afferents (GVAs) regulate food intake. Does increased maternal adiposity and maternal high‐fat, high‐sugar diet (HFHSD) reprogram offspring GVA satiety signalling?
- **What is the main finding and its importance?**
  Tension‐sensitive GVA mechanosensitivity was dampened selectively in male offspring of HFHSD dams. However, light‐phase meal size was larger in offspring of HFHSD dams regardless of sex. Therefore, dampened GVA signalling in males offers a new mechanism for intergenerational programming of obesity. In addition, the altered feeding behaviour in both sexes implies that other appetite‐regulatory pathways are also programmed by maternal HFHSD exposure.

## 1 INTRODUCTION

In 2021, an estimated 45.1% of adults aged ≥25 years worldwide were overweight or obese, with global rates predicted to increase further by 2050, from 46.7% to 60.3% in females and from 43.4% to 57.4% in males (Global Burden of Disease 2021 Adult Body Mass Index Collaborators, 2025). Although the aetiology of obesity is multifactorial (Grundy, 1998), growing evidence indicates that pregnancy is a crucial period for programming of offspring growth and lifelong health (Reynolds et al., 2022). In human populations, excessive gestational weight gain or maternal obesity increases the risk of large‐for‐gestational age offspring (Kim et al., 2014; Weschenfelder et al., 2019), and infants born large for gestational age are at increased risk of obesity (Hong & Lee, 2021) and type 2 diabetes (Knop et al., 2018) later in life. Furthermore, the positive association between maternal and offspring body mass index persists into adulthood (Zhang et al., 2024). Altered regulation of appetite in offspring might contribute to these persistent effects of exposure to maternal overweight and obesity.

Energy homeostasis and food intake are highly regulated through complex central and peripheral inputs, whilst displaying plasticity to allow for rapid adaptations in metabolic demands (Cummings & Overduin, 2007; Hopkins & Blundell, 2016; Page & Kentish, 2017). Impacts of maternal obesity on these mechanisms have been investigated in rodents. Consumption of a high‐fat diet during pregnancy and/or lactation in mice resulted in offspring that were heavier, with greater adiposity and food intake (da Silva et al., 2022). In mice and rats, feeding dams diets high in both fat and sugar [high‐fat, high‐sugar diet (HFHSD)] before and throughout pregnancy and lactation resulted in heavier offspring with greater adiposity and increased food intake after weaning (Kirk et al., 2009; Nivoit et al., 2009; Rajia et al., 2010; Samuelsson et al., 2008). Interestingly, in one of these studies, in which rat offspring of both sexes were followed to 1 year of age, effects of maternal HFHSD exposure on body weight and appetite were greater in male than female offspring (Nivoit et al., 2009).

Investigation of the mechanisms mediating hyperphagia has largely focused on hypothalamic changes and the central circuits that regulate energy homeostasis. In mice, maternal HFHSD feeding induces changes in offspring hypothalamic orexigenic [increased agouti‐related peptide (AgRP) and increased neuropeptide Y (NPY) gene and protein expression] and anorexigenic pathways [decreased proopiomelanocortin (POMC) and decreased α‐melanocyte‐stimulating hormone (α‐MSH) gene and protein expression] that would be predicted to increase food intake (da Silva et al., 2022; Dearden et al., 2020; Li et al., 2021a; Ornellas et al., 2016). Also consistent with programming of appetite‐promoting mechanisms by maternal HFHSD, young adult offspring of rat dams fed an HFHSD before and throughout pregnancy and lactation were resistant to the appetite‐suppressive effects of leptin (Kirk et al., 2009). However, juvenile and young adult male offspring of rats fed an HFHSD from the start of pregnancy until weaning only (i.e., without maternal obesity) were heavier and had more adipose tissue as adults, but with similar food intake and hypothalamic gene expression of *NPY*, *AGRP* and leptin receptor to control animals (Cesar et al., 2022). This suggests programming of additional pathways that regulate food intake.

Vagal afferent mechanosensing is an important component in the regulation of food intake. Tension‐sensitive gastric vagal afferents (GVAs) respond to distension of the stomach wall after food intake and act centrally to induce satiation (Page & Kentish, 2017; Wang et al., 2020), in addition to the initiation of feedback signals to regulate gut function, such as gastric accommodation and motility (Andrews et al., 1980; Li & Page, 2022). Mucosal GVAs respond to mucosal stroking and are thought to detect particle density and regulate gastric emptying (Becker & Kelly, 1983). Gastric vagal afferents are highly plastic, responding to circadian cues and nutritional status to control food intake finely, matching it to energy demand (Kentish et al., 2012, 2013). Increased meal size, in conditions such as obesity and pregnancy, is associated with downregulation of GVA responses to stretch in mice (Clarke et al., 2025; Kentish et al., 2012; Li et al., 2021b). In the only study to report impacts of HFHSD exposure on feeding behaviours of offspring, 5‐week‐old offspring of mice fed an HFHSD before and during pregnancy ate larger meals, regardless of sex (Kulhanek et al., 2020). Additionally, male‐specific greater meal frequency was observed in HFHSD offspring, with no effect of maternal diet on meal frequency in females, and with total food intake not reported (Kulhanek et al., 2020).

Given that meal size is, in part, a reflection of GVA function, we hypothesize that GVA responses adapt in response to maternal diet during pregnancy, leading to changes in offspring feeding behaviour. We therefore investigated the impact of maternal HFHSD feeding on offspring GVA signalling in mice, and whether there was any sex difference in the offspring exposed to this maternal dietary intervention.

## 2 MATERIALS AND METHODS

### 2.1 Ethical approval

All studies were approved by the animal ethics committee (SAM‐20‐053) of the South Australian Health and Medical Research Institute (SAHMRI), carried out in accordance with the Australian code for the care and use of animals for scientific purposes, 8th edition 2013, and adhered to the Arrive 2.0 guidelines (du Sert et al., 2018; Grundy, 2015). The authors understand the ethical principles under which the journal operates, and this research complies with these ethical principles (Grundy, 2015; O'Halloran, 2024a, 2024b).

### 2.2 Animal model

The mouse model, housing and experimental diets were as described previously (Clarke et al., 2025). Briefly, Glu Venus‐expressing mice (mice expressing yellow fluorescent protein (Venus) under the transcriptional control of the proglucagon promoter) (Reimann et al., 2008), maintained on a C57BL/6 background, were obtained under a material transfer agreement from Cambridge Enterprise Ltd, Cambridge, UK, and bred at the SAHMRI bioresources facility. The mice in this study were part of a larger study, in which the use of this strain allowed for the separation of α‐ and β‐cells within pancreatic cell populations (Reimann et al., 2008). All mice were held in the SAHMRI Bioresources facility at 22°C under 12 h–12 h light–dark cycle (lights on 07.00 h), with ad libitum access to food and water throughout the study.

Female mice (∼4 weeks old, 9–20 g) were randomized to either a standard laboratory diet (SLD; *n* = 24: Teklad standard diet: 13 kJ/g, digestible energy from protein 24%, fat 18% and carbohydrates 58%, catalogue no. 2018, Envigo, Huntingdon, UK) or a high‐fat, high‐sugar diet [HFHSD; *n* = 30: Specialty Feeds: 23 kJ/g, digestible energy from protein 17.6%, fat 58.4% (derived from soya bean and coconut oil) and carbohydrates 24% (sucrose 175 g/kg), catalogue no. SF21‐003, Glen Forrest, Western Australia, Australia] for 11.0 ± 0.4 weeks (diet phase). Female mice remained on their respective diets throughout mating, pregnancy and lactation.

During the pre‐mating phase, female mice were housed in individually ventilated cages in groups of two to five littermates and weighed weekly. Mice in both diet groups were weighed and their body composition measured using an EchoMRI Composition Analyser at 10.6 ± 0.6 weeks after starting the experimental diets (EchoMRI, Houston, TX, USA). The pre‐mating phase was designed to achieve adiposity of ≥11% in HFHSD mice. Where possible, mice in this group that had not achieved this target body fat percentage at 14 weeks of age were retained on the diet and included only if they achieved a body fat percentage of ≥11% by 17 weeks of age. Body composition data were unavailable for two SLD dams owing to equipment malfunction. The SLD mice were age‐matched, with the average age of female mice at the beginning of mating 15.1 ± 0.5 weeks of age.

Female mice were housed with a male mouse (one or two female mice per male per cage) for no more than 5 days for mating (average time to mate 2.1 ± 1.1 days) and continued to be fed their experimental diets during this period. Male mice were rotated between diet groups and replaced after ∼6 months to avoid confounding effects of paternal obesity on progeny. Female mice were checked daily at 07.00–08.00 h, and pregnancy was confirmed by the presence of a vaginal plug (assigned as gestational day 1). Plugged females were then single housed and kept on their respective diets during pregnancy and lactation.

Dams were checked daily for the presence of pups from gestational day 19, with pups remaining with dams until weaning. Litters were standardized to a maximum of five pups on postnatal day 2. Offspring mice described in the present paper were derived from 46 litters (28 SLD litters and 18 HFHSD litters), with lower numbers of HFHSD litters reflecting poorer reproductive performance.

Offspring were weaned onto the SLD at 3 weeks old and housed in ventilated cages in groups of two to five mice of the same sex and similar ages and in identical environmental conditions to the dams. Offspring were fed SLD from weaning, weighed weekly and had free access to food and water until the end of the study.

At the end of the study, mice were humanely killed by CO_2_ inhalation and cardiac puncture prior to tissue collection for electrophysiology studies. No anaesthetic was used prior to the end of the study, because no invasive procedures were performed.

### 2.3 Offspring body weight and feeding behaviour

At ∼6–7 weeks of age (46.5 ± 1.4 days old at the start of recording), one male and one female per litter, where available, were housed singly in metabolic cages (*n* = 19 male SLD, *n* = 19 female SLD, *n* = 13 male HFHSD and *n* = 13 female HFHSD) with ad libitum access to SLD and water. The offspring mice were housed singly in metabolic cages for a 2 day acclimation period, followed by a 3 day recording period, during which body weight and feeding behaviours were recorded continuously (Promethion Sable System, Las Vegas, NV, USA). Body weight, food intake, number of meals, average meal size and meal duration were analysed as previously described, with meals defined as a reduction in food hopper weight, with a minimum food intake duration of 20 s (Li et al., 2021b). Data were transformed using the Promethion data software package ExpeData version 1.9.14 (Promethion Sable System) using analytical macro 6. Body weight was averaged across all recording days, and feeding behaviours (food intake, meal size, meal duration and number of meals) were averaged overall and separately for the light and dark phases across the recording days.

### 2.4 In vitro mouse GVA electrophysiology

Tension and/or mucosal GVA recordings were obtained successfully from 41 offspring, at ∼8 weeks of age (56.6 ± 2.4 days). Given that GVA recordings could be performed on only one or two mice per day, these recordings included only 10 of the mice from which metabolic cage data were obtained. Mice were humanely killed between 07.00 and 07.30 h by CO_2_ inhalation and cardiac puncture. The electrophysiological methods used to record mouse GVA activity have been described in detail previously (Li et al., 2013; Page et al., 2002a). Briefly, the thorax was opened to remove the stomach and oesophagus, and the vagal nerves were separated from the oesophagus. The stomach was opened with the vagal nerves attached and placed mucosal side up in an organ bath filled with a modified Krebs solution, including nifedipine (1 µm) to prevent smooth muscle contraction. The vagal nerves were placed into another chamber, filled with liquid paraffin. The nerves were teased apart into small bundles and placed onto a platinum recording electrode for single‐fibre recording. Nerve impulses were amplified (DAM50, World Precision Instruments, Sarasota, FL, USA), filtered (band‐pass filter 932, CWE, Ardmore, PA, USA) and recorded.

GVA mechanosensitivity was identified by locating receptive fields on the stomach, where tension‐sensitive GVAs respond to mucosal stroking and tension stimuli, whereas mucosal GVAs respond to mucosal stroking only (Page et al., 2002b). To record the responses of tension‐sensitive afferents to stretch, a threaded hook was attached adjacent to the receptive field and to a cantilever system. Tension stimuli were created by placing weights (0.5–5 g) on the cantilever system for 1 min. To record the responses of mucosal afferents, the receptive field was stroked with calibrated von Frey hairs (10–1000 mg). Up to five individual tension‐sensitive or mucosal afferents were recorded per mouse. Action potentials of single units were analysed using Spike 2 software (Cambridge Electronic Design, UK). When recordings were obtained from more than one GVA subtype in an individual mouse, data were averaged to create one data point per GVA subtype per mouse.

### 2.5 Statistical analysis

The Levine test was used to evaluate homogeneity of variance between groups, and the Shapiro–Wilk test to evaluate normality within each group. Where these assumptions were not met, non‐parametric tests were used. Effects of diet on maternal body weight before mating were analysed by one‐way ANOVA. Effects of diet on maternal body composition were assessed by the Mann–Whitney *U*‐test. Meal duration was not normally distributed, and effects of maternal diet on this outcome were therefore analysed by the Mann–Whitney *U*‐test separately within each progeny sex. Effects of maternal diet and progeny sex on all other offspring outcomes obtained from metabolic cage recordings, where a maximum of one male and one female mouse were included per litter, were analysed by two‐way ANOVA. Offspring GVA data were analysed by repeated‐measures mixed model. Models assessed the impact of maternal diet, offspring sex and load (von Frey hair weight or tension), and treated measures made on the same offspring as repeated measures. Owing to limited animal availability, it was necessary to collect GVA data from up to two males or two females from some litters, and these models therefore included the identity of the dam as a random factor to correct for effects of the common maternal environment. Statistical analyses were conducted using SPSS version 28 (IBM Corporation, Armonk, NY, USA), and data are presented as the mean ± SD unless otherwise stated. Interaction terms are reported only where *p* < 0.1.

## 3 RESULTS

### 3.1 Maternal phenotype before mating

**FIGURE 1:**
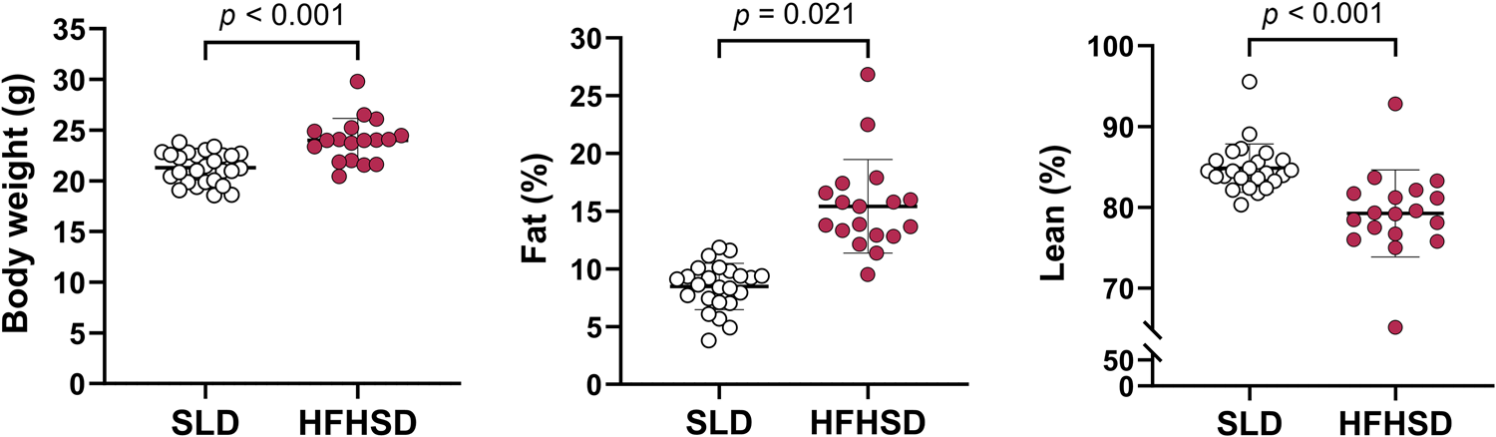
Maternal body weight and body composition before mating. Body weight was obtained for 27 SLD and 18 HFHSD dams, and body composition for 25 SLD and 18 HFHSD dams. Effects of diet on maternal body weight were assessed by one‐way ANOVA, and effects on maternal body composition were assessed by the Mann–Whitney *U*‐test. Symbols indicate data for individual mice. Bars and whiskers indicate the mean ± SD for each group. Abbreviations: HFHSD, dams fed a high‐fat, high‐sugar diet for 11 weeks before mating and throughout pregnancy and lactation; SLD, dams fed a standard laboratory diet for 11 weeks before mating and throughout pregnancy and lactation.

Prior to mating, HFHSD females were 13% heavier, with a 66% higher fat mass relative to body weight and 5% lower relative lean mass, in comparison to SLD females (Figure 1).

### 3.2 Offspring body weight and feeding behaviour

**FIGURE 2:**
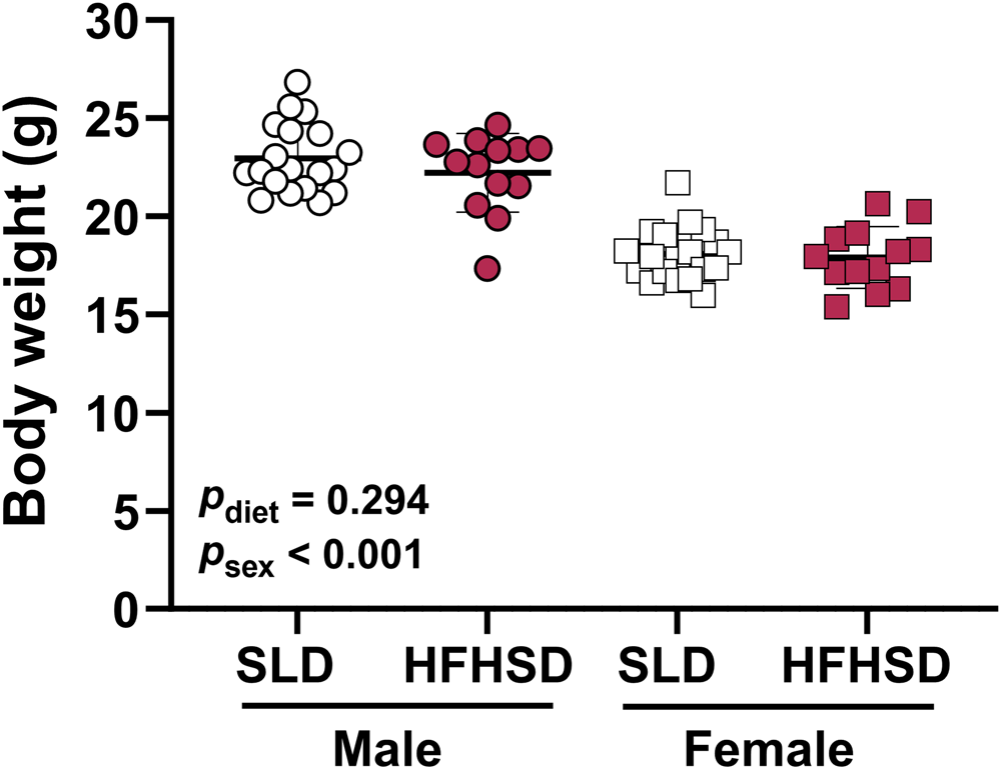
Offspring body weight during metabolic cage recordings at 6–7 weeks of age. Effects of maternal diet and progeny sex were assessed by two‐way ANOVA (*n* = 19 SLD male offspring, *n* = 13 HFHSD male offspring, *n* = 19 SLD female offspring and *n* = 13 HFHSD female offspring). Symbols indicate data for individual mice. Bars and whiskers indicate the mean ± SD for each group. Abbreviations: HFHSD, offspring of dams fed a high‐fat, high‐sugar diet for 11 weeks prior to mating and throughout pregnancy and lactation; SLD, offspring of dams fed a standard laboratory diet for 11 weeks prior to mating and throughout pregnancy and lactation.

Maternal diet did not impact offspring weight measured during metabolic cage recordings, and male offspring were heavier than female offspring (Figure 2).

**FIGURE 3:**
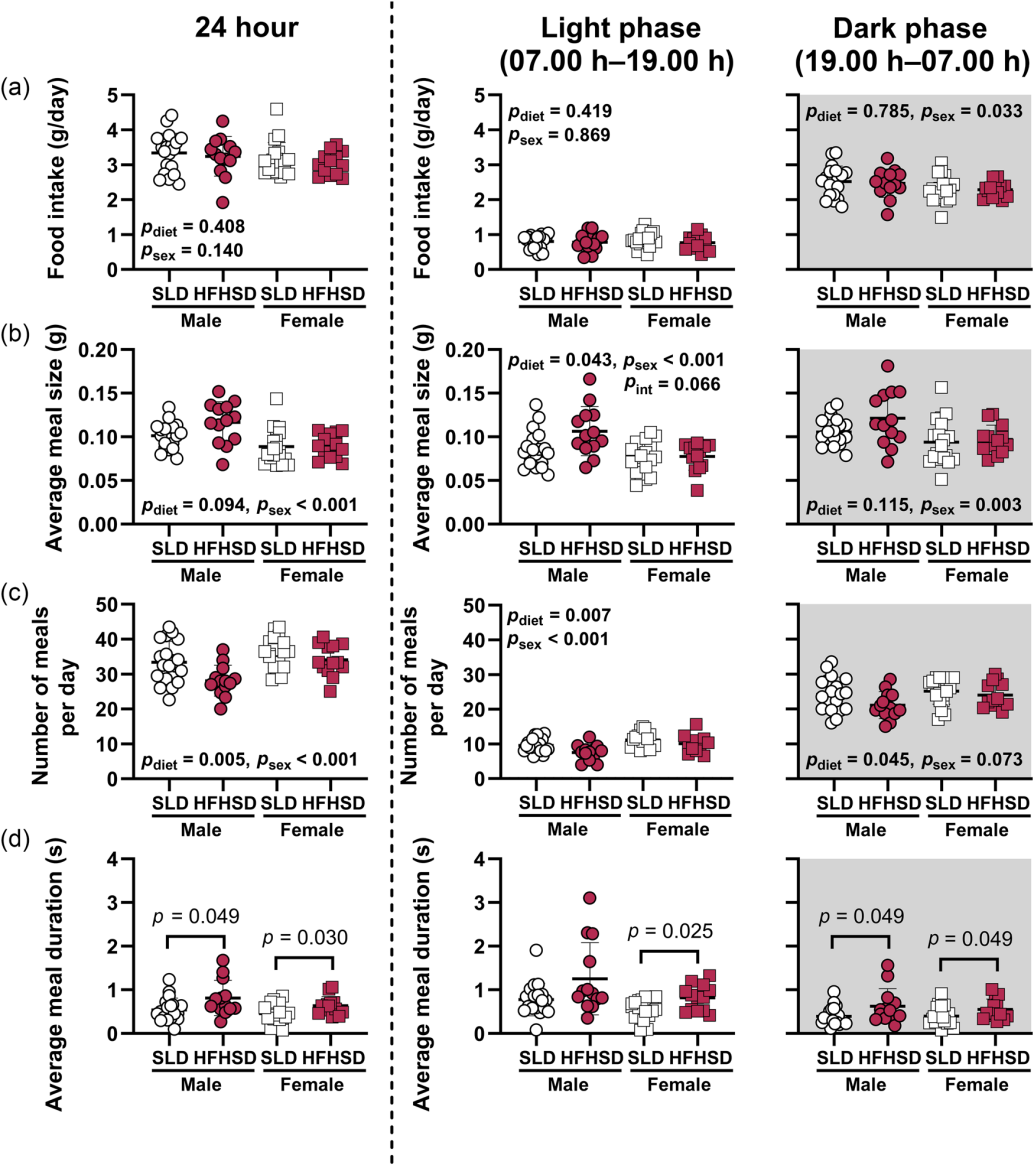
Offspring feeding behaviour during metabolic cage recordings at 6–7 weeks of age. Effects of maternal diet and progeny sex on food intake (a), meal size (b) and number of meals (c) were assessed by two‐way ANOVA (*n* = 19 SLD male offspring, *n* = 13 HFHSD male offspring, *n* = 19 SLD female offspring and *n* = 13 HFHSD female offspring). Effects of maternal diet on meal duration (d) were assessed separately by the Mann–Whitney *U*‐test within male and female offspring. Symbols indicate data for individual mice. Bars and whiskers indicate the mean ± SD for each group. Abbreviations: HFHSD, offspring of dams fed a high‐fat, high‐sugar diet for 11 weeks prior to mating and throughout pregnancy and lactation; int, maternal diet × offspring sex interaction; SLD, offspring of dams fed a standard laboratory diet for 11 weeks prior to mating and throughout pregnancy and lactation.

Total food intakes across 24 h and during the light phase were not different in offspring from each maternal diet group, nor between male and female offspring (Figure 3a). Food intake relative to body weight across 24 h and during the light phase did not differ between maternal diet groups (*p* = 0.711 and *p* = 0.500, respectively) and were higher in female than male offspring (each *p* < 0.001). Absolute food intake during the dark phase was 9% higher in male than female offspring, but also unaffected by maternal diet (Figure 3a). In contrast, dark phase food intake relative to body weight did not differ between maternal diet groups (*p* = 0.698) and was higher in female than male offspring (*p* < 0.001). Average meal sizes across 24 h and during the dark phase were similar in offspring of SLD and HFHSD dams and were 18%–20% larger in male than female mice (Figure 3b). However, during the light phase, average meal sizes of HFHSD offspring were 14% larger than those of SLD offspring and were 22% larger in males than females (Figure 3b). HFHSD offspring ate fewer meals across 24 h, and during both light and dark phases, than SLD offspring (Figure 3c). Male offspring ate fewer meals than females across 24 h and during the light phase, but the number of meals during the dark phase did not differ between sexes (Figure 3c). Meal duration was non‐normally distributed and was therefore analysed separately using non‐parametric tests in male and female offspring. Meal duration averaged across 24 h and in the dark phase was longer in HFHSD than SLD within both males and females (Figure 3d). Meal duration in the light phase was longer in HFHSD females than SLD females but did not differ between HFHSD and SLD males (Figure 3d).

### 3.3 In vitro mouse gastric vagal afferent electrophysiology

**FIGURE 4:**
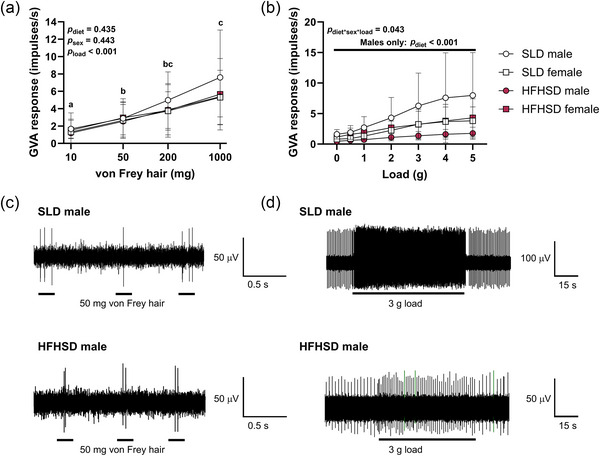
Gastric vagal afferent responses in offspring at 8 weeks of age. (a, b) Mucosal (a) and/or tension‐sensitive (b) afferent responses were obtained from 12 SLD male offspring, 11 HFHSD male offspring, 10 SLD female offspring and 8 HFHSD female offspring. (c, d) Representative recordings from male offspring of SLD and HFHSD dams are shown for mucosal GVAS in response to 50 mg stroking (c) and tension‐sensitive GVAs in response to 3 g tension (d). Bars indicate application of load. Electrophysiological data were analysed by mixed model for effects of maternal diet, offspring sex and load, treating outcomes on each progeny as repeated measures. Owing to a significant three‐way interaction and non‐normally distributed data, effects of maternal diet on tension‐sensitive GVA responses were assessed separately by the Mann–Whitney *U*‐test within male and female offspring. Symbols and whiskers indicate the mean ± SD for each group. Letters (a, b, c) indicate differences between loads. Abbreviations: GVA, gastric vagal afferent; HFHSD, offspring of dams fed a high‐fat, high‐sugar diet for 11 weeks prior to mating and throughout pregnancy and lactation; *p*_diet*sex*load_, maternal diet × offspring sex × load interaction; SLD, offspring of dams fed a standard laboratory diet for 11 weeks prior to mating and throughout pregnancy and lactation.

The response of mucosal afferents to stroking was unaffected by maternal diet or offspring sex and increased with load (Figure 4a). Mucosal responses were greater at 50, 200 and 1000 mg than at 10 mg (*p* = 0.038, *p* < 0.001 and *p* < 0.001, respectively) and were greater at 1000 mg than at 50 mg (*p* = 0.003). Representative responses of mucosal GVAs to stroking with a 50 mg von Frey hair in male offspring of SLD and HFHSD dams are illustrated (Figure 4c).

Effects of maternal diet and offspring sex on responses of tension‐sensitive GVAs differed with load (three‐way interaction, *p* = 0.043). Tension responses within each load were not normally distributed, and we therefore used Mann–Whitney *U*‐tests to analyse effects of maternal diet within male and female offspring separately at each load. In male offspring, the firing frequency of tension‐sensitive GVAs was lower in offspring of HFHSD dams than in offspring of SLD dams at all loads, from 0 to 5 g (each *p* < 0.001; Figure 4b). In female offspring, the firing frequency of tension‐sensitive GVAs did not differ between diet groups at any load (0 g, *p* = 0.408; 0.5 g, *p* = 0.142; 1 g, *p* = 0.351; 2 g, *p* = 0.606; 3 g, *p* = 1.000; 4 g, *p* = 0.758; and 5 g, *p* = 1.000; Figure 4b). Representative responses of tension‐sensitive GVAs to a 3 g load in male offspring of SLD and HFHSD dams are illustrated (Figure 4d).

## 4 DISCUSSION

Our data demonstrate male‐specific programming of GVA responses to stretch, resulting from in utero and lactation exposure to elevated maternal adiposity and continued HFHSD consumption. Intriguingly, although GVA responses to stretch were dampened only in males, offspring of both sexes consumed larger meals during the light phase. These changes in feeding behaviour suggest that the maternal HFHSD also programs other satiety mechanisms, particularly in females. Downregulation of GVA satiety mechanisms did not translate to increased food intake or body weight in these adolescent offspring, although these might develop with age.

Despite impacts on GVA responses to tension and feeding behaviour, maternal HFHSD feeding that induced elevated maternal adiposity at mating and was continued throughout pregnancy and lactation did not affect offspring body weight at 6–7 weeks of age, nor offspring food intake in the present study. Others have likewise reported in C57BL/6J mice that body weights are similar in offspring of lean control and obese HFHSD‐fed dams soon after weaning, although HFHSD offspring become heavier with ageing and can have more adipose tissue than control animals even at ages when weights are similar (Dearden et al., 2020; Kulhanek et al., 2020; Samuelsson et al., 2008; Schoonejans et al., 2022). Nevertheless, the impacts of maternal HFHSD exposure on offspring are somewhat variable even between studies using the same strain of mouse (Nicholas & Ozanne, 2019), possibly reflecting the different maternal diets used. Furthermore, HFHSD exposure has different impacts on male and female offspring in some cases. For example, both male and female offspring of obese C57BL/6J mice fed a high‐fat, high‐sugar cafeteria diet (58% fat and 25.5% sucrose) for 12 weeks prior to mating and through pregnancy and lactation were heavier at weaning than control offspring (King et al., 2013). However, in the same study, offspring weights at 3–5 months of age were unaffected by maternal diets, and at 6 months of age female HFHSD offspring were lighter than control offspring, with no weight difference between HFHSD and control males (King et al., 2013). Conversely, male‐specific heavier body weight has been reported in two different HFHSD models. In C57BL/6J mice, feeding high‐fat chow supplemented with sweetened condensed milk to female mice for 10 weeks, from weaning and through their first pregnancy and lactation, resulted in maternal obesity, hyperglycaemia and glucose intolerance in a second, experimental pregnancy, during which HFHSD feeding was continued (Schoonejans et al., 2022). Both male and female offspring from this second pregnancy and lactation had greater fat mass than control animals by 5–6 months of age, but only males became heavier than control animals, although not until 9 months of age (Schoonejans et al., 2022). In a slightly different model, in which the duration of pre‐mating feeding was shorter (6 weeks; 58% fat plus sucrose HFHSD), male but not female HFHSD offspring were heavier than control animals by weaning, and this persisted to adulthood (Park et al., 2020).

Evidence from the limited number of studies in which food intake has been measured suggests that greater caloric intake accompanies or precedes weight excess in offspring of HFHSD dams. For example, offspring of mice fed an HFHSD (16% fat and 33% sugar) for 6 weeks before mating and throughout pregnancy and lactation had greater caloric intake than control offspring from 4 weeks of age in males and from 6 weeks of age in females; differences in weight were significant in males from 12 weeks of age and were transient in 5‐ to 11‐week‐old females, before re‐emerging at 22 weeks of age (Samuelsson et al., 2008). Dearden et al. (2020) reported similar food intake in male HFHSD and control offspring at 6 and 8 weeks of age and did not measure food intake in females. Effects of maternal diet on food intake might emerge at later ages, given that the weight of HFHSD males exceeds that of control animals by 9 months of age in this model (Dearden et al., 2020). The lack of difference in food intake at 6 months of age between control and HFHSD male offspring in another study is likely to reflect limited power (*P* = 0.0508, *n* = 3–8 per group), given that the HFHSD males in that study were heavier than control males from 3 weeks of age (Park et al., 2020). In the sole study to report weight deficits in HFHSD mouse offspring, which was specific to females, daily food intakes at 6 months of age were similar in control and HFHSD offspring of both sexes (King et al., 2013). Exposure to maternal obesity and HFHSD throughout pregnancy and lactation likewise induces higher feed intakes and higher body weights in Wistar and Sprague–Dawley rats, with impacts seen in both male and female offspring (Kirk et al., 2009; Nivoit et al., 2009). Although in the present study, offspring food intake was not impacted by elevated maternal adiposity and continued HFHSD feeding during pregnancy and lactation, this exposure changed the feeding behaviour of both male and female offspring, increasing meal size in the light phase and meal duration in the dark phase, and decreasing the number of meals in both light and dark phases. Additionally, light‐phase meal duration was lower in HFHSD than SLD, in female offspring only. Only one other study has reported the impacts of maternal HFHSD consumption on offspring feeding behaviour. In offspring of mice fed HFHSD (40% fat plus sucrose) for 4–6 weeks before and throughout pregnancy and lactation, 4‐ to 5‐week‐old male HFHSD offspring ate larger and more frequent meals than control male offspring, with no differences in feeding behaviour between female offspring groups, although weight and adiposity differences were not yet evident in either sex (Kulhanek et al., 2020). Data in that study was not reported separately for light and dark periods, hence the impacts of HFHSD exposure on circadian patterns of behaviour could not be assessed. Together, these studies suggest that maternal obesity with pregnancy and lactation HFHSD exposure usually upregulates the appetite of offspring, although the age at which this emerges can vary. Longitudinal studies are needed to determine whether the altered feeding behaviour seen in our cohort at ∼7 weeks of age is followed by hyperphagia and weight gain.

Given that GVAs play an important role in the short‐term regulation of food intake, particularly meal size and meal frequency (Page & Kentish, 2017), we also assessed GVA responses to stretch and stroke stimuli in the offspring of control and HFHSD‐fed dams. This is the first study to investigate the effects of consumption of an HFHSD, ‘Western’‐style diet, before and during pregnancy and lactation on peripheral satiety pathways. We observed a sex‐specific desensitization of GVAs in male offspring of HFHSD dams, adding to our recent report that chronic HFHSD consumption reduced GVA sensitivity to stretch in non‐pregnant adult female mice (Clarke et al., 2025). One potential mechanism for male‐specific programming of lower offspring GVA tension signalling might be through adiponectin, which selectively reduced gastric vagal afferent responses to stretch, with no effect on mucosal afferents in mice (Kentish et al., 2015). In rats, plasma levels of adiponectin were higher in male, but not female, offspring from dams fed a high‐fat diet compared with control diet (Gregoraszczuk et al., 2016). Whether male‐specific elevated adiponectin is present in offspring of HFHSD dams and could contribute to reduced GVA responses in mice requires further investigation.

In theory, desensitization of GVAs should lead to an increase in meal size, as a consequence of extended meal duration, given that GVA satiety signals terminate a meal (Berthoud, 2008). Indeed, meal size was larger in HFHSD offspring than SLD offspring regardless of sex, predominantly owing to an increase in meal size during the light phase. This was reflected by an increase in meal duration in the light and dark phase in female mice and only in the dark phase in male mice, with high variability in meal duration during the light phase in male mice. This lower GVA mechanosensitivity was observed in tissue collected at the beginning of the light phase (∼07.00 h), consistent with desensitization of tension‐sensitive GVAs in HFHSD male offspring contributing to their greater light‐phase meal size. The lack of change in GVA responses in HFHSD compared with control female offspring suggests that other appetite‐regulatory pathways must also be involved. Others have reported hypothalamic changes in HFHSD progeny in conjunction with greater appetite. Specifically, HFHSD male and female offspring had downregulation of hypothalamic anorexigenic signals and upregulation of orexigenic signals, relative to control progeny (da Silva et al., 2022; Dearden et al., 2020; Li et al., 2021a; Ornellas et al., 2016). Whether hypothalamic changes also contribute to the greater meal size in female offspring in the present model of HFHSD requires further investigation. In addition to studies of these central satiety pathways, studies of other peripheral satiety pathways, including small intestinal responses to stretch and nutrients and the impacts of cholecystokinin and leptin on vagal afferent signals, would add further understanding of the mechanisms underlying intergenerational obesity.

## 5 CONCLUSION

In conclusion, tension‐sensitive GVA satiety signalling is dampened by in utero and lactation exposure to elevated maternal adiposity and continued maternal HFHSD consumption, but in males only. However, meal size was greater in HFHSD offspring in both sexes, suggesting that other appetite‐regulatory pathways must also be programmed by the maternal HFHSD, especially for the female offspring. Dampened GVA satiety signalling concurrent with greater meal size suggests a new mechanism for the intergenerational programming of obesity by the consumption of a Western diet before and during pregnancy and lactation.

## AUTHOR CONTRIBUTIONS

Lisa M. Nicholas established and provided access to the experimental model of HFHSD‐feeding during pregnancy in the Glu Venus mice. All authors contributed to experimental design. Stephanie E. O'Hara, Kelly M. Gembus, Georgia S. Clarke, Hui Li and Elaheh Heshmati conducted experiments. Kathryn L. Gatford and Georgia S. Clarke analysed data. Kathryn L. Gatford, Amanda J. Page and Georgia S. Clarke wrote the manuscript. All authors contributed to interpretation of data and editing of the manuscript. All authors approved the final version of the manuscript and agree to be accountable for all aspects of the research in ensuring that questions related to the accuracy or integrity of any part of the work are appropriately investigated and resolved. All individuals designated as authors qualify for authorship and all those who qualify for authorship are listed.

## CONFLICT OF INTEREST

No conflicts of interest, financial or otherwise, are declared by the authors.

## DATA AVAILBILITY STATEMENT

Data will be made available upon reasonable request.
